# Immobilized Native Bacteria as a Tool for Bioremediation of Soils and Waters: Implementation and Modeling

**DOI:** 10.1100/tsw.2002.211

**Published:** 2002-05-18

**Authors:** C. Lobo, M. Sanchez, C. Garbi, E. Ferrer, M.J. Martinez-Iñigo, J.L. Allende, C. Martinez, L. Casasas, R. Alonso, A. Gibello, M. Martin

**Affiliations:** Facultad de Veterinaria, Universidad Complutense, 28040 Madrid, Spain; Inst. Madrileño de Investigacion Agraria y Alimentaria. Apt. 127, 28800 Madrid, Spain; ETSIA y ETSII, Universidad Politécnica, 28040 Madrid, Spain

**Keywords:** bioremediation, biofilm, bacteria, molecular techniques, modeling, bioreactor, aromatic compounds

## Abstract

Based on 3,4-dihydroxyphenylacetate (3,4-DHPA) dioxygenase amino acid sequence and DNA sequence data for homologous genes, two different oligonucleotides were designed. These were assayed to detect 3,4-DHPA related aromatic compound—degrading bacteria in soil samples by using the FISH method. Also, amplification by PCR using a set of ERIC primers was assayed for the detection of *Pseudomonas* GCH1 strain, which used in the soil bioremediation process. A model was developed to understand and predict the behavior of bacteria and pollutants in a bioremediation system, taking into account fluid dynamics, molecular/cellular scale processes, and biofilm formation.

### INTRODUCTION

The efficient application of bioremediation projects calls for a monitoring of the microorganisms involved in the degradation or removal of target pollutants at remediation sites. Aromatic compound–degrading bacteria are most often used in these bioremediation systems, because aromatic compounds are particularly stable and prevalent in nature. They enter the environment through both natural processes and human industrial and agricultural pursuits.

We have designed bioremediation systems using immobilized bacteria that can degrade different pollutant compounds such as 4-hydroxyphenylacetate, 4-hydroxybenzoate, propachlor and oxadiazone[1,2]. Some of the genes involved in these degradative pathways have been cloned and the enzymes characterized[3,4,5].

In the present study, based on the DNA sequence data of genes involved in ring cleavage, two different oligonucleotides were designed, then assayed to detect aromatic compound–degrading bacteria in soil samples by using the FISH method. We have also tested selective PCR primers for the detection of bacteria used in soil bioremediation processes.

Besides the general aim, this contribution gives account of the modeling of the complex phenomena involved in the proposed bioremediation systems.

### EXPERIMENTAL METHODS

#### Bacterial Strains, Plasmids, and Conditions of Growth

*Escherichia coli* CC118 (pAG464) was obtained by transformation of *E. coli* CC118 with pAG464, a derivative of pUC18 plasmid containing the *Klebsiella pneumoniae hpa*B gene[3]. Cells were grown aerobically at 37ºC in LB medium and Ampicillin (Ap) was added at the final concentration of 100 mg/ml. *Pseudomonas* GCH1[2] was used in the soil bioremediation assays. *Pseudomonas aeruginosa*, an aromatic-degradative strain was assayed in FISH experiments.

#### Soil Microcosms

Microcosms consisted of trays in which were placed 700 g of an alkaline loam soil (pH 8.2, 12% clay, 46% sand, 42% silt) with organic matter and nitrogen percentages of 0.86% and 0.065%, respectively. Soils were inoculated with LB liquid culture of GCH1 bacterial strain at the exponential phase of growth. After a 10-day incubation at 25°C and 60% air humidity, DNA was extracted from 500-mg soil samples using the FastDNA spin kit for soil (BIO 101, Carlsbad, CA).

#### PCR Assays

PCR was performed in a total volume of 25 mL. The final reaction mixture contained 1 unit of Ecotaq DNA polymerase (Ecogen, S.R.L.), deoxynucleoside triphosphates at a concentration of 400 mM, 1x Ecotaq buffer and 2 mM $Mg_2Cl$ solution. The repetitive intergenic consensus (ERIC) sequences were used to design PCR primers[6]. Before amplification, samples were heated at 94ºC for 5 min. Then they were subjected to 36 cycles of denaturation at 94ºC for 1 min, annealing at 52ºC for 1 min and extension at 72ºC for 2 min, followed by a final extension at 72ºC for 5 min. The 25 mL reaction was separated by 2% agarose gel electrophoresis and the products visualised by UV light after ethidium bromide staining.

#### FISH

FISH assays were carried out at 43ºC, according to Du Teau et al.[7]. Slides were examined on a Confocal Laser Scanning Microscopy MRC 1024 Biorad.

## RESULTS AND DISCUSSION

### Validation of the Designed DHPA Diox Oligonucleotides

Computer analysis of the *K. pneumoniae* DNA fragment cloned in pAG464 revealed an open reading frame (ORF), corresponding to the *hpa* B gene, that starts at position 798 bp, encoding the DHPA diox, which showed a high overall identity with its homologous enzymes of *E. coli,* strains C and W[8,9], and *Salmonella dublin* (DHPA Sd: accession number AF144422). Since the comparison of these enzymes to the DHPA diox from *P. aeruginosa* (DHPA Pa)[10] also revealed a high grade of identity (63%), this type of enzyme seems to be highly conserved at least among these enterobacterial species and *Pseudomonas* sp. This comparison suggests a common evolutionary origin for the DHPA diox in gram-negative bacteria very different to that established for the homologous enzymes isolated from gram-positive bacteria belonging to the major extradiol dioxygenase family.

So based on the comparison of the DHPA diox amino acid sequence with homologous aromatic dioxygenases and according the scheme shown in Fig.1, we have designed two different oligonucleotide probes for use in FISH assays:

Diox 1: 5´ - GTGGTNTCGNTCTCCGC - 3´
Diox 2: 5´ - TCNGCCTTCTGTACCGTT - 3´

The applicability of the developed probes to detect aromatic compound–degrading bacteria was confirmed by the FISH method for two selected representative strains, *E. coli* CC118(pAG464) and *P. aeruginosa*. The specific detection of the *hpa* gene was demonstrated for both probes. This molecular technique has shown that designed oligonucleotides are suitable for detecting bacteria with specific metabolic capabilities.

### GCH1 Strain Identification by PCR Assays

Fig. 2 shows the specific DNA amplification patterns for pure GCH1 culture in LB liquid medium. When DNA was extracted from soil samples inoculated with GCH1 strain, the PCR pattern shared three fragments (800 pb, 1100 pb, and at between 1500 and 2072 bp-size fragments) and exhibited an extra fragment of 500 pb (Fig. 2, lanes c,d) that is most likely due to amplification products from soil-native microorganism DNA.

The results indicate that ERIC sequences permit the identification of pesticide-degrading bacteria within the soil microbial population. Consequently, specific PCR are proposed for monitoring the bacteria introduced in contaminated soils submitted to remediation by bioaugmentation techniques.

### Modeling the Bioremediation Process

To understand and predict the behavior of bacteria and pollutants in the proposed bioremediation system we give a formulation in terms of three differential equations:

$$\dot{s} = -\mu_s(t)x(t) - mx(t) + F(t) + cx^2(t) + v(S(t)-s(t))$$

$$\dot{x} = \mu_x(t)x(t) - cx^2(t) + D(t)$$

$$\dot{S} = rv(s(t) - S(t))$$

where s(t) is the substrate (pollutant) concentration in the bioreactor, x(t) the size of the population and S(t) the concentration of substrate in the tank.

These equations are a modified version of the Chemostat Model capable of giving account of the observed phenomena in the lag, exponential and stationary phases of population growth. The coeffi-

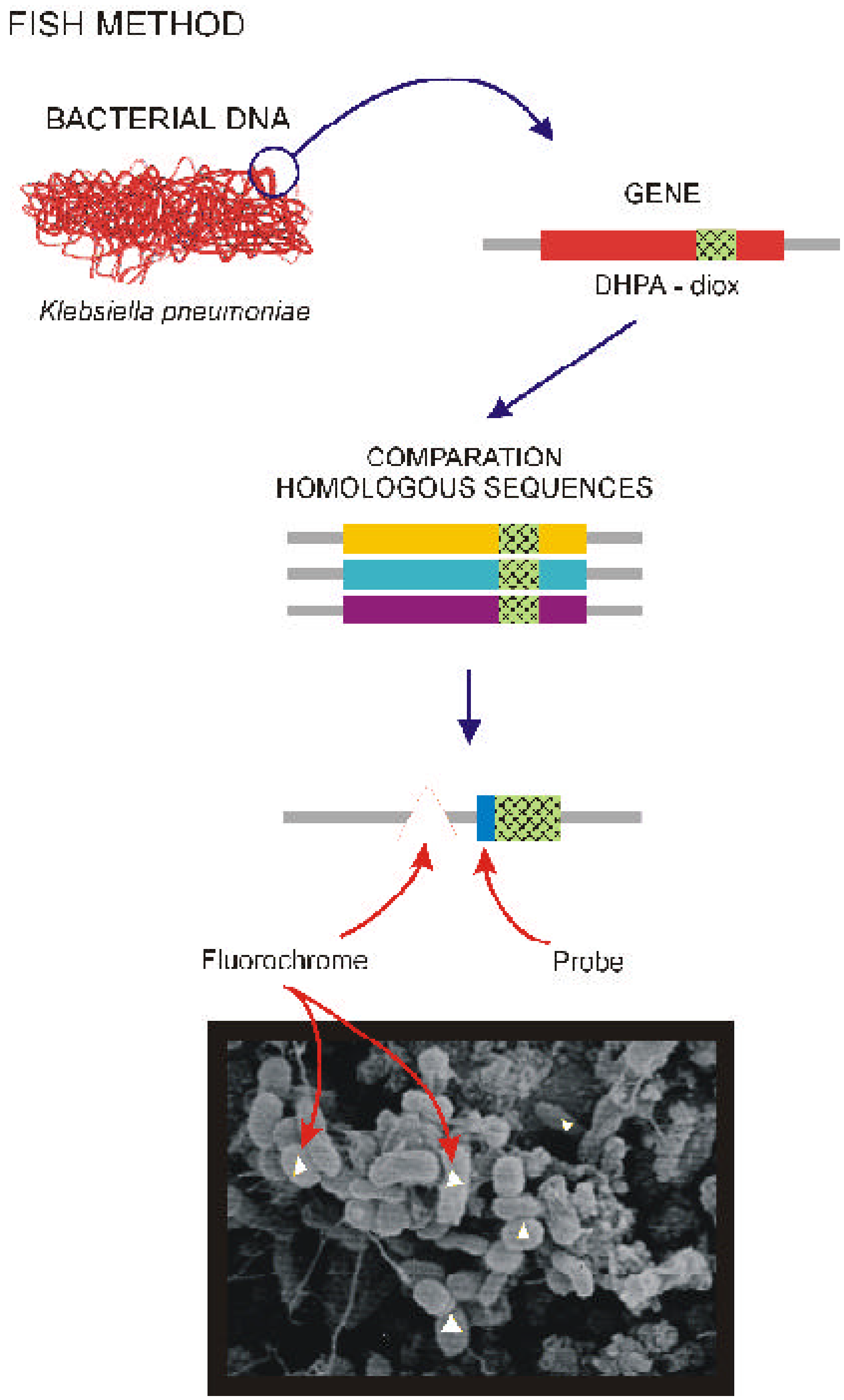

**FIGURE 1.** The FISH method: selective molecular probes based on 3,4-dihydroxyphenylacetate dioxigenase (DHPA diox), were assayed to detect aromatic compounds–degrading bacteria in soil samples.

cients are defined as follows:

$k_s$ = saturation constant (Michaelis-Menten)

m = maintenance factor

$$\mu_x(t) = \frac{s(t) - a(t)s(t)^2}{s(t) + k_s}$$

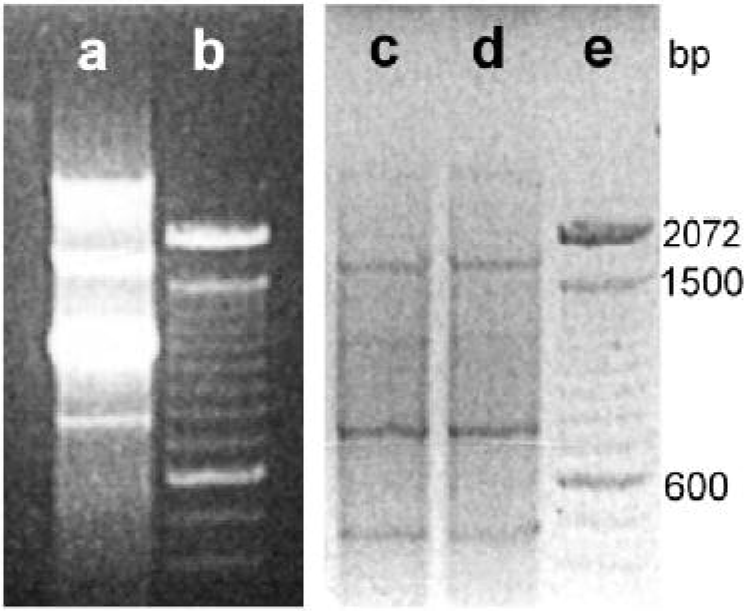

**FIGURE 2.** PCR results obtained by using ERIC sequences as primers for the amplification of the genomic DNA from GCH1 strain isolated from: (Lane a) pure bacterial culture in LB liquid medium; (Lanes c, d) soil samples inoculated with GCH1 at different doses; (Lanes b, e) 100 bp DNA ladder marker (GibcoBRL) (2 µL).

$$\mu_s(t) = \frac{s(t)}{s(t) + k_s}$$

$$a(t) = a\,(1 + b\exp(-gt))$$

F(t) : extra input (co-substrate)

D(t) : biomass losses v: tank-bioreactor flow r: (bioreactor volume)/(tank volume)

In our equations we take a linear growth term and a quadratic death term. We also consider what happens in the case of a learning process in order to consume a new substrate and also the global effects of poisoning and substrate inputs/outputs. This is achieved through the coefficient $\mu_x(t)$, which includes a quadratic term in s(t) and the maximum nonpoisonous concentration given by

$$s_{max}(t) = \frac{1}{a(t)}.$$

Note that when the substrate concentration rises to this value, the growth factor $\mu_x(t)$ is zero; a and b are constants determining the rate of the learning process.

Some of the advantages of this model follow from the knowledge of the function a(t). In Fig. 3 we present the results of a simulation in which the flow tank/bioreactor is modified according to the tolerance developed by bacteria towards increasing pollutant concentrations. In particular,

$$v(t) = 0.002 + \frac{0.06t}{1200 + t}$$

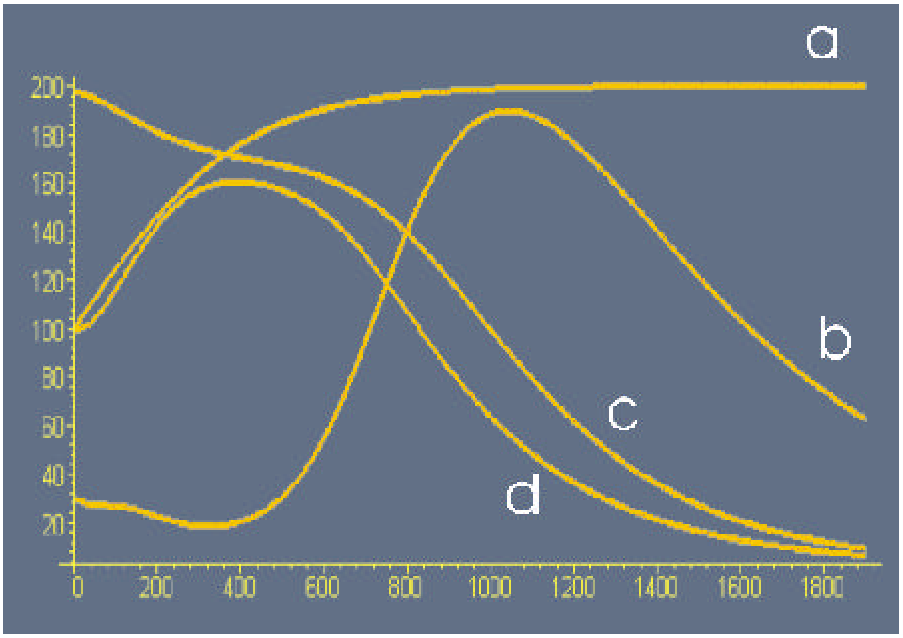

**FIGURE 3.** Substrate metabolization: (a) toxic substrate concentration, (b) biomass, (c) influent substrate concentration, (d) bioreactor substrate concentration.

so that is maintained close to $s_{max}(t)$. As a result of this regulation of v(t), the charge of pollutant in the tank can be as large as S(0) = 2s(0). In fact, the value of S(t) at the end of the operation is 1/5 of the concentration obtained by just using a constant flow v(t) = 0.002. This represents a clear improvement in the efficiency of the bioremediation.

An auxiliary tool in the construction of this model is an accurate knowledge of the processes at the cellular aggregate scale. More precisely, the coefficients in the differential equations are taken form the local space-time distribution of the biomass u(x,t) described by an integrodifferential equation:

$$u_t = \mathbf{c}(u)u_{xx} + \mathbf{b}u - \mathbf{d}u^2 + \mathbf{m}(x,t)$$

This reaction-diffusion equation models the temporal change in cell density at each point *x*. We consider two classes of supports:

1. A microsphere of alginate, where x = 0 represents the surface of the ball and x = L its center.
2. A granulus of sepiolite, where x = 0 is the outer surface of the biofilm and x = L the maximum depth.

The diffusion coefficient c = c(u) depends on the size of the aggregate. The local growth rate

$$b(u,x,t) = \frac{1}{A + B\int_0^x u(r,t)dr}$$

is modified by the amount of population in [0,x] i.e.,

$$\int_0^x u(r,t)dr \,.$$

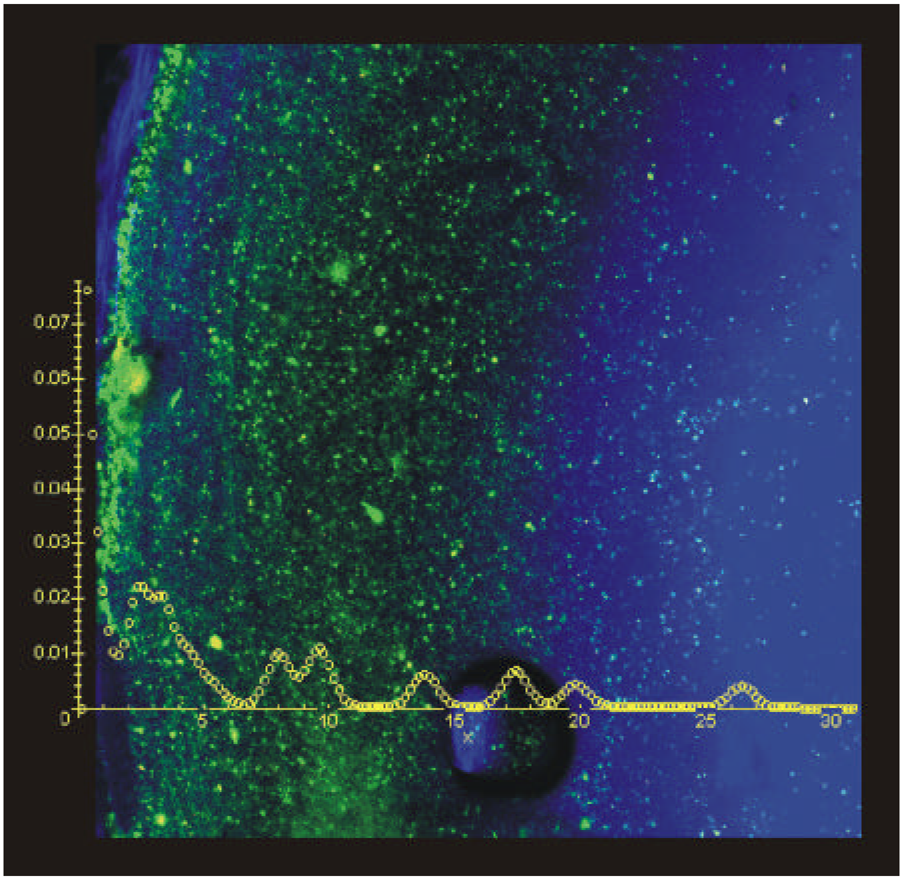

**FIGURE 4.** Biofilm: distribution of cells into alginate beads.

In doing this, we assume that the substrate is available through diffusion from the exterior of the support and will be first consumed by the microbes closer to the surface.

The term m(x,t) is a random variable giving account of the cell migration from the aggregates and transport of microbes by the fluid. The observed spatial distribution of cellular aggregates in alginate is reproduced in the numerical experiments as in Fig. 4, corresponding to a simulation with a random initial distribution.

### CONCLUSIONS

The work presented in this paper has shown that molecular techniques are effective tools for identifying bacteria in environmental samples, that PCR method that utilizes ERIC probes is an effective tool for the detection of specific bacterial strains used in bioremediation processes, and that the FISH method is an useful system for the evaluation of natural attenuation potential in environmental samples. The proposed model permits the knowledge of microscopic processes to be incorporated, allowing for a fairly accurate description and control of the global bioremediation methodology.

### ACKNOWLEDGEMENTS

This work was partly supported by grant FP-01-46 from IMIA (Comunidad de Madrid). This paper was presented at the CSIC/ESF workshop “Analysis, Toxicity and Biodegradation of Organic Pollutants in Groundwater from Contaminated Land, Landfills and Sediments" Barcelona, Spain, November 8–10, 2001.
